# Cross-Omics Identification and Validation of PCSK9 as a Candidate Molecule Associated with Aortic Dissection and Negative Remodeling After TEVAR

**DOI:** 10.3390/jcdd13090442

**Published:** 2026-09-07

**Authors:** Sheng Fang, Chenghao Yang, Jincheng Fang, Yong Ding, Xu Li, Tiancheng Xie, Zhenyu Zhou, Min Zhou, Zhenyu Shi

**Affiliations:** 1 Department of Vascular Surgery, Zhongshan Hospital, Institute of Vascular Surgery, Fudan University, Shanghai 200032, China; 23111210152@m.fudan.edu.cn (S.F.); 24111210174@m.fudan.edu.cn (C.Y.);; 2 National Clinical Research Center for Interventional Medicine, Shanghai 200032, China

**Keywords:** type B aortic dissection, TEVAR, PCSK9, aortic remodeling, cross-omics

## Abstract

**Background:** Negative aortic remodeling after thoracic endovascular aortic repair (TEVAR) remains a major challenge in patients with type B aortic dissection (TBAD). This exploratory study aimed to integrate human aortic tissue transcriptomic data with serum proteomic profiles from a remodeling-stratified TEVAR cohort to prioritize candidate molecules associated with TBAD and negative aortic remodeling. **Methods:** Public human aortic tissue transcriptomic data from GSE190635 were analyzed to identify differentially expressed genes between aortic dissection and control aortic tissues. Serum tandem mass tag (TMT)-based untargeted proteomic profiling was performed in a remodeling-stratified TEVAR cohort, including patients with negative remodeling and those with stable or positive remodeling. Cross-omics integration was conducted by intersecting differentially expressed genes and differentially abundant serum proteins. The prioritized candidate molecule was further validated using serum ELISA, quantitative real-time PCR, Western blotting, hematoxylin and eosin staining, immunohistochemistry, and immunofluorescence co-staining in human serum and aortic tissue samples. **Results:** Transcriptomic analysis identified 1534 differentially expressed genes in aortic dissection tissues, while serum proteomic profiling identified 82 differentially abundant proteins associated with postoperative remodeling status. Cross-omics integration revealed seven overlapping molecules, among which PCSK9 was the only candidate showing concordant upregulation in both aortic dissection tissues and serum samples from patients with negative remodeling after TEVAR. Serum PCSK9 levels were significantly higher in patients with aortic dissection than in normal controls. PCSK9 mRNA and protein expression were also increased in human aortic dissection tissues. Histological and immunostaining analyses showed enhanced PCSK9 accumulation in the dissected aortic wall, particularly in medial regions with partial overlap with α-SMA-positive areas. **Conclusions:** PCSK9 was identified as a cross-omics prioritized candidate molecule associated with aortic dissection and negative remodeling after TEVAR. Its elevation in serum and aortic tissues, together with its enrichment in α-SMA-positive medial regions, suggests a potential link between circulating proteomic alterations and local aortic wall pathology. These findings support further investigation of PCSK9 as a candidate biomarker for adverse remodeling in TBAD.

## 1. Introduction

Type B aortic dissection (TBAD) is a complex vascular disorder in which disruption of the intimal layer initiates sustained hemodynamic and biological remodeling of the aortic wall [1]. Thoracic endovascular aortic repair (TEVAR) has become the standard treatment for complicated TBAD because it seals the proximal entry tear, redirects blood flow into the true lumen, and promotes false lumen thrombosis [2,3]. However, favorable early technical outcomes do not always translate into durable aortic remodeling. Persistent false lumen perfusion, incomplete thrombosis, distal re-entry flow, progressive aortic dilatation, and late reintervention remain clinically important challenges after TEVAR [4,5,6].

The aortic media is central to dissection progression and post-TEVAR remodeling. Under physiological conditions, VSMCs maintain vascular tone and extracellular matrix homeostasis, whereas inflammatory, oxidative, and mechanical stress can drive VSMCs toward maladaptive phenotypes [7,8,9]. Loss of contractile identity, enhanced proteolytic activity, extracellular matrix degradation, and inflammatory crosstalk contribute to medial weakening and progressive aortic dilatation. Molecules linking systemic circulating signals with local medial injury may therefore be particularly relevant for understanding adverse remodeling in TBAD.

Proprotein convertase subtilisin/kexin type 9 (PCSK9) is best known for its role in low-density lipoprotein receptor degradation and cholesterol metabolism [10,11]. Beyond lipid regulation, accumulating evidence indicates that PCSK9 may also participate in vascular inflammation, endothelial dysfunction, macrophage activation, oxidative stress, and vascular remodeling [12,13,14]. These non-lipid vascular effects make PCSK9 biologically relevant to vascular diseases characterized by inflammatory activation, extracellular matrix instability, and VSMC dysfunction.

Single-layer discovery approaches may be insufficient to capture the biological complexity of TBAD after TEVAR. Transcriptomic profiling of human aortic tissue can identify local disease-associated molecular programs, whereas serum proteomic profiling can reveal circulating proteins associated with postoperative remodeling phenotypes. Integrating these complementary layers may help prioritize molecules that are both biologically connected to local aortic pathology and accessible through peripheral blood sampling [15,16]. In this study, we combined public human aortic tissue transcriptomic analysis, serum proteomic profiling from a remodeling-stratified TEVAR cohort, and validation in human serum and aortic tissue samples to identify candidate molecules associated with TBAD and adverse aortic remodeling after TEVAR.

## 2. Materials and Methods

### 2.1. Study Design

This study was designed as an integrated translational biomarker discovery and validation study to identify candidate molecules associated with type B aortic dissection (TBAD) and negative aortic remodeling after thoracic endovascular aortic repair (TEVAR). The overall workflow consisted of four sequential components: public human aortic tissue transcriptomic analysis, serum proteomic profiling in a remodeling-stratified cohort of TBAD patients undergoing TEVAR, cross-omics integration of transcriptomic and proteomic data, and orthogonal validation using human serum and aortic tissue samples. Public transcriptomic analysis and serum proteomics served as discovery layers, whereas serum- and tissue-based experiments were performed as validation layers.

### 2.2. Public Aortic Tissue Transcriptomic Dataset and Differential Expression Analysis

The public transcriptomic dataset GSE190635 was obtained from the Gene Expression Omnibus database [17]. This dataset was generated using the GPL570 Affymetrix Human Genome U133 Plus 2.0 Array platform and contains bulk microarray expression profiles of human aortic tissue samples. The dataset included ascending aortic tissues from four male patients with acute Stanford type A aortic dissection and four male healthy controls. Dissection tissues were collected during Bentall operations, whereas control aortic tissues were obtained from healthy organ donors without cardiovascular diseases.

The processed expression matrix and corresponding platform annotation file were downloaded from GEO. Probe identifiers were mapped to official gene symbols according to the GPL570 annotation file [18]. Probes without matched gene symbols were removed. When multiple probes mapped to the same gene symbol, the probe with the highest mean expression value across all samples was retained for downstream analysis.

Differential expression analysis between aortic dissection and control aortic tissue samples was performed using the limma package in R [19]. Linear models were fitted for each gene, followed by empirical Bayes moderation. For exploratory screening, differentially expressed genes were defined as genes with a nominal *p* value < 0.05 and an absolute log2 fold change > 1, corresponding to more than two-fold upregulation or downregulation.

### 2.3. Clinical Cohort, Aortic Remodeling Assessment, and Proteomic Discovery Cohort Selection

From December 2020 to October 2021, patients who underwent TEVAR for TBAD at the Department of Vascular Surgery, Zhongshan Hospital, Fudan University, were selected as the study cohort. Eligible patients were those with TBAD confirmed by aortic computed tomography angiography (CTA), successful standard TEVAR treatment, and complete preoperative and postoperative imaging and hematological data. Patients were excluded if they had malignant tumors, severe infection, autoimmune disease, a history of previous open aortic surgery, hereditary connective tissue disease such as Marfan syndrome, severe hepatic or renal dysfunction, or extreme preoperative hemodynamic instability such as shock. Patients were also excluded if intraoperative angiography revealed an untreated endoleak, or if the first postoperative CTA follow-up, such as at 1 or 3 months, confirmed the presence of any type of endoleak.

Postoperative aortic remodeling was assessed using CTA images obtained approximately 12 months after TEVAR. Based on the Society for Vascular Surgery classification criteria for aortic remodeling after TEVAR [3,20], positive aortic remodeling was defined by any of the following morphological changes: false lumen thrombosis with a reduction in total aortic diameter of more than 5 mm at the maximal aortic diameter, unchanged total aortic diameter with an increased true lumen diameter, or unchanged total aortic diameter with a decreased false lumen diameter. Negative aortic remodeling was defined as a decrease in true lumen volume and/or an increase in false lumen volume, or an increase in aortic diameter of more than 5 mm or more than 10% compared with the preoperative measurement. CTA images were independently reviewed by two vascular surgeons experienced in aortic disease imaging assessment (S.F. and M.Z.). Both reviewers were blinded to serum PCSK9 levels, proteomic results, and subsequent statistical analyses. In cases of disagreement, the final classification was determined after reassessment by a third senior vascular specialist (ZY.S.).

Peripheral venous blood samples were collected from all included patients before TEVAR. According to the 12-month postoperative CTA-based remodeling assessment, patients with negative remodeling were assigned to the progression group (P group), whereas those with stable or positive remodeling were assigned to the stable group (S group). Using the computer random number method, 6 serum samples from the progression group and 6 from the stable group are randomly selected respectively to form the “Discovery cohort” (P6 vs. S6), which is used for the subsequent non-targeted proteomics sequencing.

This study was approved by the Ethics Committee of Zhongshan Hospital Affiliated to Fudan University (approval number: B2019-110). Written informed consent for clinical data use and biological sample collection was obtained from all included patients or their legal representatives.

### 2.4. Serum Collection and TMT-Based Untargeted Proteomic Profiling

Peripheral venous blood samples were collected before TEVAR and processed for serum isolation. Serum samples were aliquoted and stored at −80 °C until proteomic analysis, and repeated freeze–thaw cycles were avoided.

For the proteomic discovery cohort, six serum samples from the progression group and six serum samples from the stable group were analyzed. TMT-based untargeted proteomic profiling was performed by Shanghai Luming Biotechnology Co., Ltd. (Shanghai, China). Briefly, high-abundance serum proteins were depleted, and protein concentration was determined using a BCA assay. Equal amounts of protein from each sample were digested with trypsin, labeled with TMTpro reagents, pooled, fractionated by high-pH reversed-phase liquid chromatography, and analyzed by liquid chromatography–tandem mass spectrometry using an EASY-nLC 1000 system coupled to an Orbitrap Fusion mass spectrometer [21,22]. All samples were processed within the same TMTpro-based multiplexed proteomic workflow, including identical downstream fractionation and LC-MS/MS procedures, to minimize potential analytical batch effects. Raw mass spectrometry data were processed using Proteome Discoverer software version 2.4.1.15 and searched against the UniProt Homo sapiens reference proteome database. Protein identification was controlled at a false discovery rate of less than 1%. Relative protein abundance was quantified based on TMT reporter ion intensities [23,24]. Differentially abundant proteins between the progression and stable groups were defined as proteins with fold change ≥ 1.2 or ≤1/1.2 and *p* value < 0.05.

### 2.5. Cross-Omics Integration of Transcriptomic and Proteomic Data

Differentially expressed genes from the public aortic tissue transcriptomic dataset and differentially abundant serum proteins from the TMT-based proteomic analysis were integrated using official gene symbols. Gene and protein identifiers were standardized before integration, and entries without valid gene symbols were excluded.

Shared molecules between the two datasets were identified by intersecting the transcriptomic and proteomic candidate lists. For each overlapping molecule, the gene symbol, transcriptomic fold change, transcriptomic *p* value, proteomic fold change, proteomic *p* value, and direction of change were summarized. The overlap was visualized using a Venn diagram, and the integrated candidate list was used for subsequent validation analyses.

### 2.6. Human Serum and Aortic Tissue Validation

Human serum and aortic tissue samples were used for orthogonal validation of the integrated candidate molecule. To evaluate circulating PCSK9 levels, serum samples from patients with TBAD were obtained from the previously established clinical cohort before TEVAR. In addition, healthy volunteers were recruited as normal controls (NC group) from the Health Examination Center of Zhongshan Hospital, Fudan University. Individuals in the NC group were excluded if they had a history of aortic disease or aortic surgery, or if they had severe acute or chronic infection, hepatic or renal dysfunction, or malignant tumors.

To assess the expression and localization of PCSK9 at the local tissue level, human aortic dissection tissues and non-dissected control aortic tissues were collected. Because TBAD is primarily treated with endovascular repair and diseased aortic wall specimens are difficult to obtain, aortic dissection tissues were collected from patients who underwent open aortic graft replacement for acute Stanford type A aortic dissection at the Department of Cardiac Surgery, Zhongshan Hospital, Fudan University. All dissection specimens were obtained from the ascending aorta. Normal control aortic samples were sourced from the ascending aorta of heart transplant donors. Tissue samples were either immediately stored at −80 °C for molecular assays or fixed in 4% paraformaldehyde, embedded in paraffin, and sectioned for histological and immunostaining analyses.

Serum PCSK9 concentrations were measured using a Human PCSK9 ELISA Kit (MM-14341H1, Jiangsu Meimian Industrial Co., Ltd., Yancheng, China) according to the manufacturer’s instructions. Briefly, serum samples and standards were added to ELISA plates and incubated with the corresponding detection reagents. After washing, substrate solution was added, and the reaction was stopped according to the protocol. Absorbance was measured at 450 nm using a microplate reader, and PCSK9 concentrations were calculated from the standard curve.

For molecular validation in human aortic tissues, total RNA was extracted from the suprarenal aorta using Trizol Reagent (Invitrogen, Carlsbad, CA, USA) according to the manufacturer’s protocol. A 1 mg sample of total RNA of the aorta was reverse-transcribed to cDNA using Evo M-MLVMix Kit with gDNA Clean for qPCR (AG11728, Accurate Biology, Changsha, China). The mRNA levels of PCSK9 and GAPDH in human aortic samples were measured by qRT-PCR using SYBRGreen Premix Pro Taq HS qPCR Kit (AG11718, Accurate Biology, Changsha, China) and QuantStudio 5 Real-Time PCR System according to the manufacturer’s protocol. The relative mRNA expression of PCSK9 was calculated using the 2^−ΔΔCt^ method [25,26], with GAPDH as the endogenous control. The primer sequences were as follows: human PCSK9, forward 5′-GAGTGGGACATCACAGGCTG-3′ and reverse 5′-GGGAACCAGGCCTCATTGAT-3′; human GAPDH, forward 5′-GCACCGTCAAGGCTGAGAAC-3′ and reverse 5′-TGGTGAAGACGCCAGTGGA-3′. Total proteins were extracted from human aortic tissues using RIPA buffer (Beyotime Institute of Biotechnology, Shanghai, China) supplemented with protease inhibitors. After quantification, protein samples (10–20 μg) were separated on 10% SDS-PAGE gels (PG212, Epizyme, Shanghai, China) and transferred onto polyvinylidene fluoride (PVDF) membranes (Millipore, Burlington, MA, USA). The membranes were blocked with 5% non-fat milk in Tris-buffered saline containing 0.1% Tween-20 (TBST) for 1 h at room temperature and then incubated overnight at 4 °C with the following primary antibodies: anti-PCSK9 antibody (1:1000; Proteintech, 55206-1-AP) and anti-β-actin antibody (1:5000; Proteintech, 20536-1-AP). After washing, the membranes were incubated with HRP-conjugated AffiniPure goat anti-rabbit IgG secondary antibody (1:5000; Proteintech, SA00001-2). Protein bands were visualized using ECL Prime Western Blotting Detection reagent and quantified using ImageJ software (version 1.54p; National Institutes of Health, Bethesda, MD, USA).

For histological and immunostaining analyses, paraffin-embedded human aortic tissue sections were deparaffinized, rehydrated, and stained with hematoxylin and eosin according to standard procedures. For immunohistochemistry, sections were subjected to antigen retrieval, endogenous peroxidase blocking, and non-specific binding blockade, followed by overnight incubation at 4 °C with an anti-PCSK9 primary antibody (1:200, Proteintech, 55206-1-AP). The sections were then incubated with a universal HRP-polymer secondary antibody for mouse/rabbit primary antibodies (G1303, Servicebio, Wuhan, China). Immunoreactivity was visualized using a DAB substrate kit (G1202, Servicebio, Wuhan, China), and nuclei were counterstained with hematoxylin. IHC images were acquired under identical magnification and acquisition settings. Comparable medial regions of the aortic wall were selected, and three non-overlapping high-power fields were analyzed per specimen. DAB-positive staining was quantified using ImageJ with the Color Deconvolution plugin. The integrated optical density and ROI area were measured, and AOD was calculated as integrated optical density divided by ROI area. All images were coded before analysis, and quantification was performed by an investigator blinded to group allocation [27,28].

For immunofluorescence co-staining, sections prepared as described above were incubated overnight at 4 °C with primary antibodies against PCSK9 (1:200; Proteintech, 55206-1-AP) and α-SMA (1:200; Abcam, ab7817). After washing, the sections were incubated with species-appropriate fluorescent secondary antibodies (Invitrogen, USA), and nuclei were counterstained with DAPI. Images were captured using a confocal microscope. The localization of PCSK9 in α-SMA-positive vascular smooth muscle cell-rich regions was assessed in representative fields.

### 2.7. Statistical Analysis

Statistical analyses were performed using R software version 4.5.2 and GraphPad Prism version 9.5.1 (GraphPad Software, San Diego, CA, USA). Continuous variables were assessed for normality using the Shapiro–Wilk test. Data are presented as mean ± standard deviation (SD) and were compared using an unpaired two-tailed Student’s *t*-test or Mann–Whitney U test, as appropriate. Categorical variables were compared using Fisher’s exact test. Differences were considered statistically significant when *p*  <  0.05.

## 3. Results

### 3.1. Cross-Omics Analysis Identified PCSK9 as a Candidate Molecule Associated with Aortic Dissection and Negative Remodeling After TEVAR

**Figure 1:**
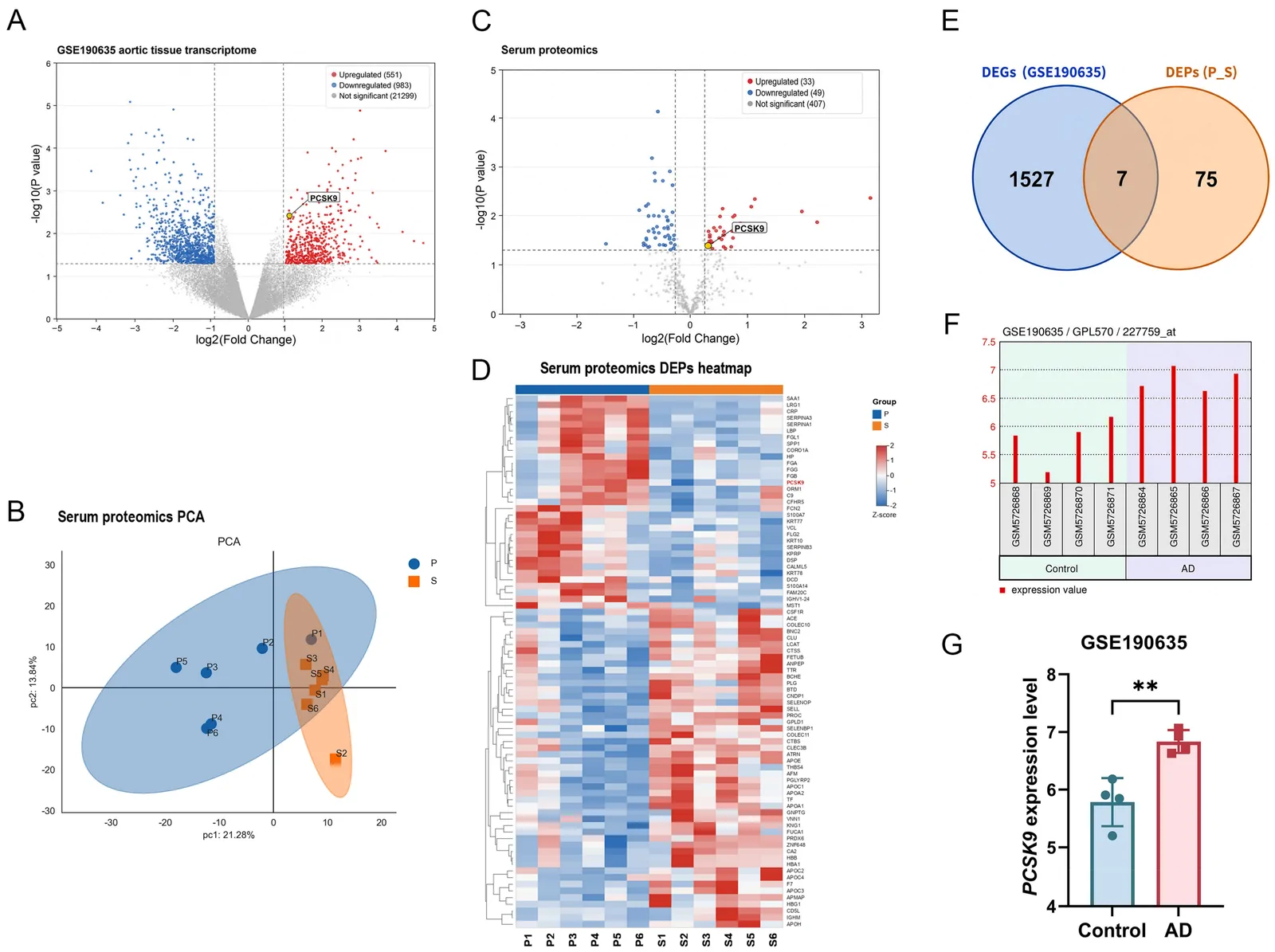
Integrated transcriptomic and serum proteomic analyses identified PCSK9 as a candidate molecule associated with aortic dissection and negative remodeling after TEVAR. (**A**) Volcano plot showing DEGs between AD and control aortic tissue samples in the GSE190635 dataset. Red and blue dots indicate upregulated and downregulated genes, respectively, and gray dots indicate non-significant genes. PCSK9 is highlighted. (**B**) Principal component analysis (PCA) of serum proteomic profiles from patients with negative remodeling and those with stable or positive remodeling after TEVAR (*n* = 6). Blue and orange indicate the progression (P) and stable (S) groups, respectively. (**C**) Volcano plot showing DEPs between the P and S groups. Red and blue dots indicate upregulated and downregulated proteins, respectively, whereas gray dots indicate non-significant proteins. (**D**) Heatmap of the 82 DEPs identified by serum proteomic analysis. Rows represent proteins and columns represent individual serum samples. PCSK9 is marked in the heatmap. Red and blue indicate relatively higher and lower protein abundance, respectively, based on Z-score normalization; the column annotation colors indicate the P and S groups. (**E**) Venn diagram showing the overlap between DEGs from the GSE190635 dataset and DEPs from the serum proteomic analysis. Seven shared molecules were identified. (**F**) Individual expression values of PCSK9 probe 227759_at in the GSE190635 dataset. (**G**) Quantitative comparison of PCSK9 expression between control and AD aortic tissue samples in GSE190635. Data are shown as mean ± SD. ** *p* < 0.01 vs. Control. AD, aortic dissection; DEGs, differentially expressed genes; DEPs, differentially abundant proteins; P, progression/negative remodeling group; S, stable or positive remodeling group; TEVAR, thoracic endovascular aortic repair.

To identify genes associated with aortic dissection at the local tissue level, we first analyzed the public human aortic tissue transcriptomic dataset GSE190635, which included aortic wall samples from four patients with aortic dissection and four control subjects. Using a threshold of *p* < 0.05 and |log2 FC| > 1, a total of 1534 differentially expressed genes were identified between aortic dissection and control aortic tissues, including 551 upregulated and 983 downregulated genes (Figure 1A). The full list of differentially expressed genes is provided in Supplementary Table S1. No gene met the BH-FDR < 0.05 threshold in GSE190635. Therefore, the nominally screened genes were used only for exploratory candidate prioritization and not interpreted as FDR-confirmed differential-expression findings.

We next performed serum proteomic profiling in a remodeling-stratified TBAD cohort after TEVAR. Six serum samples from patients with negative remodeling and six serum samples from patients with stable or positive remodeling were selected for TMT-based untargeted proteomic analysis. The available baseline characteristics of the proteomic discovery cohort are summarized in Supplementary Table S2. Given the limited sample size, these data are descriptive and do not establish covariate balance. Residual confounding by measured and unmeasured clinical factors cannot be excluded. Principal component analysis showed partial separation between the two groups, suggesting distinct serum proteomic profiles associated with postoperative remodeling phenotypes (Figure 1B). Differential abundance analysis identified 82 differentially abundant serum proteins between the progression and stable groups, including 33 upregulated and 49 downregulated proteins in the progression group (Figure 1C). The full list of differentially abundant serum proteins is provided in Supplementary Table S3. Hierarchical clustering of these differentially abundant proteins further showed distinct expression patterns between the two remodeling groups (Figure 1D).

To integrate local aortic tissue transcriptomic alterations with circulating proteomic changes, differentially expressed genes from GSE190635 and differentially abundant serum proteins from the TEVAR cohort were intersected using official gene symbols. The Venn diagram showed seven overlapping molecules between the 1534 differentially expressed genes and the 82 differentially abundant serum proteins (Figure 1E). Detailed information on these shared molecules, including SERPINA1, APOA1, IGHM, FGA, ORM1, S100A7, and PCSK9, is summarized in Table 1. A one-sided hypergeometric test based on the common measurable molecular background showed that the observed seven-molecule overlap did not represent a statistically significant enrichment beyond that expected by chance (*p* = 0.3739). Therefore, the cross-omics intersection was interpreted as an exploratory candidate-reduction step rather than evidence of statistically significant cross-omics enrichment.

The seven overlapping molecules were not assigned a formal statistical rank. Most candidates either showed discordant directions of change between the transcriptomic and proteomic layers or were downregulated in both datasets. PCSK9 was the only shared molecule showing concordant upregulation in both aortic dissection tissue at the transcriptomic level and serum from patients with negative remodeling after TEVAR at the proteomic level. PCSK9 was therefore prioritized for focused downstream validation based primarily on this cross-omics directional concordance, together with its suitability for orthogonal validation in serum and aortic tissue and its previously reported relevance to vascular inflammation and remodeling. Fold changes and *p* values for the seven candidates are reported descriptively in Table 1 and were not used to generate a formal rank order. To further visualize the transcriptomic alteration of PCSK9 in aortic tissues, PCSK9 expression values were extracted from the GSE190635 dataset and analyzed separately. At the individual sample level, PCSK9 expression was higher in aortic dissection tissues than in control aortic tissues (Figure 1F). Quantitative comparison further showed significantly increased PCSK9 mRNA expression in the aortic dissection group (Figure 1G).

### 3.2. PCSK9 Was Upregulated in Human Serum and Aortic Dissection Tissues

**Figure 2:**
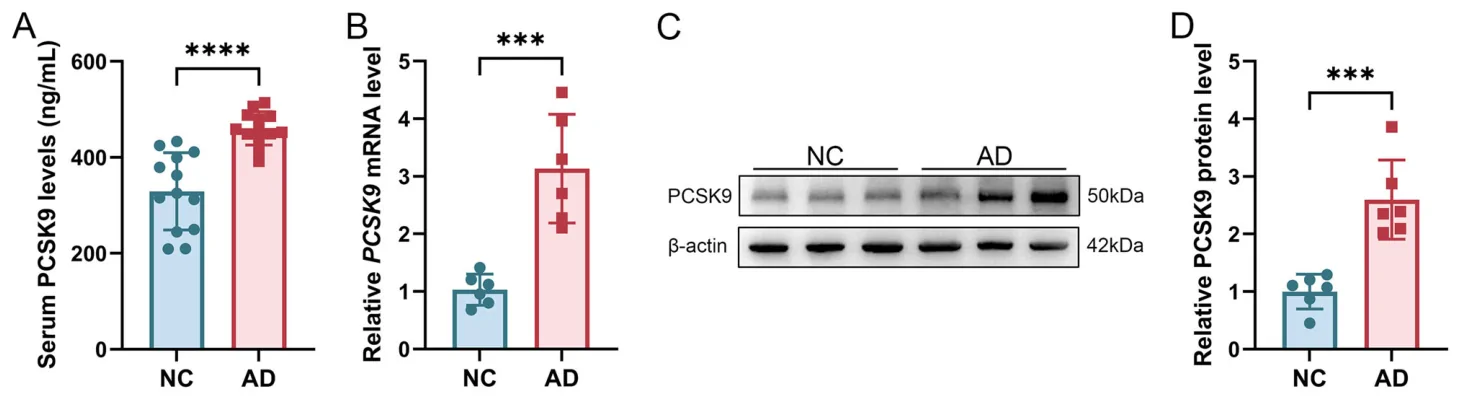
PCSK9 was upregulated in human serum and aortic dissection tissues. (**A**) Serum PCSK9 concentrations measured by ELISA in normal control (NC) subjects and patients with aortic dissection (AD). (**B**) Relative PCSK9 mRNA expression in NC and AD aortic tissue samples, as determined by quantitative real-time PCR. PCSK9 expression was normalized to GAPDH and expressed relative to the NC group. (**C**) Representative Western blot images showing PCSK9 protein expression in NC and AD aortic tissues, with β-actin used as the loading control. (**D**) Densitometric quantification of PCSK9 protein levels normalized to β-actin and expressed relative to the NC group. Data are shown as mean ± SD, and each point represents an individual sample (*n* = 6). Statistical comparisons were performed using an unpaired two-tailed Student’s *t*-test or Mann–Whitney U test, as appropriate. *** *p* < 0.001 and **** *p* < 0.0001 vs. NC. AD, aortic dissection; NC, normal control; PCSK9, proprotein convertase subtilisin/kexin type 9, ELISA, enzyme-linked immunosorbent assay.

After PCSK9 was prioritized by the integrated transcriptomic and serum proteomic analysis, we further validated its expression in independent human serum and aortic tissue samples. Serum PCSK9 concentrations were measured by ELISA. Compared with the NC group, patients in the AD group showed significantly higher circulating PCSK9 levels (460.4 ± 34.7 ng/mL vs. 329.1 ± 80.5 ng/mL; *p* < 0.0001; Figure 2A), supporting the peripheral detectability of PCSK9 and its association with aortic dissection.

We next examined whether PCSK9 was also upregulated in human aortic tissue. Quantitative real-time PCR analysis showed that PCSK9 mRNA expression was significantly increased in AD tissues compared with NC aortic tissues (Figure 2B), corresponding to an approximately 3.04-fold increase in the AD group. This finding was consistent with the transcriptomic signal observed in the GSE190635 dataset and indicated that PCSK9 was transcriptionally upregulated in the local diseased aortic wall. To further determine whether the increased transcript level was accompanied by increased protein expression, Western blotting was performed in human aortic tissue samples. Representative immunoblots showed stronger PCSK9 bands in AD tissues than in NC tissues, with β-actin used as the loading control (Figure 2C). Densitometric analysis confirmed that the relative PCSK9 protein level was significantly higher in the AD group (2.60 ± 0.69 vs. 1.00 ± 0.30; *p* = 0.0004; Figure 2D).

Together, these serum and tissue validation results indicate that PCSK9 is consistently elevated at the circulating, mRNA, and protein levels in association with aortic dissection.

### 3.3. PCSK9 Was Enriched in Aortic Dissection Tissues and Localized to α-SMA-Positive Medial Regions

**Figure 3:**
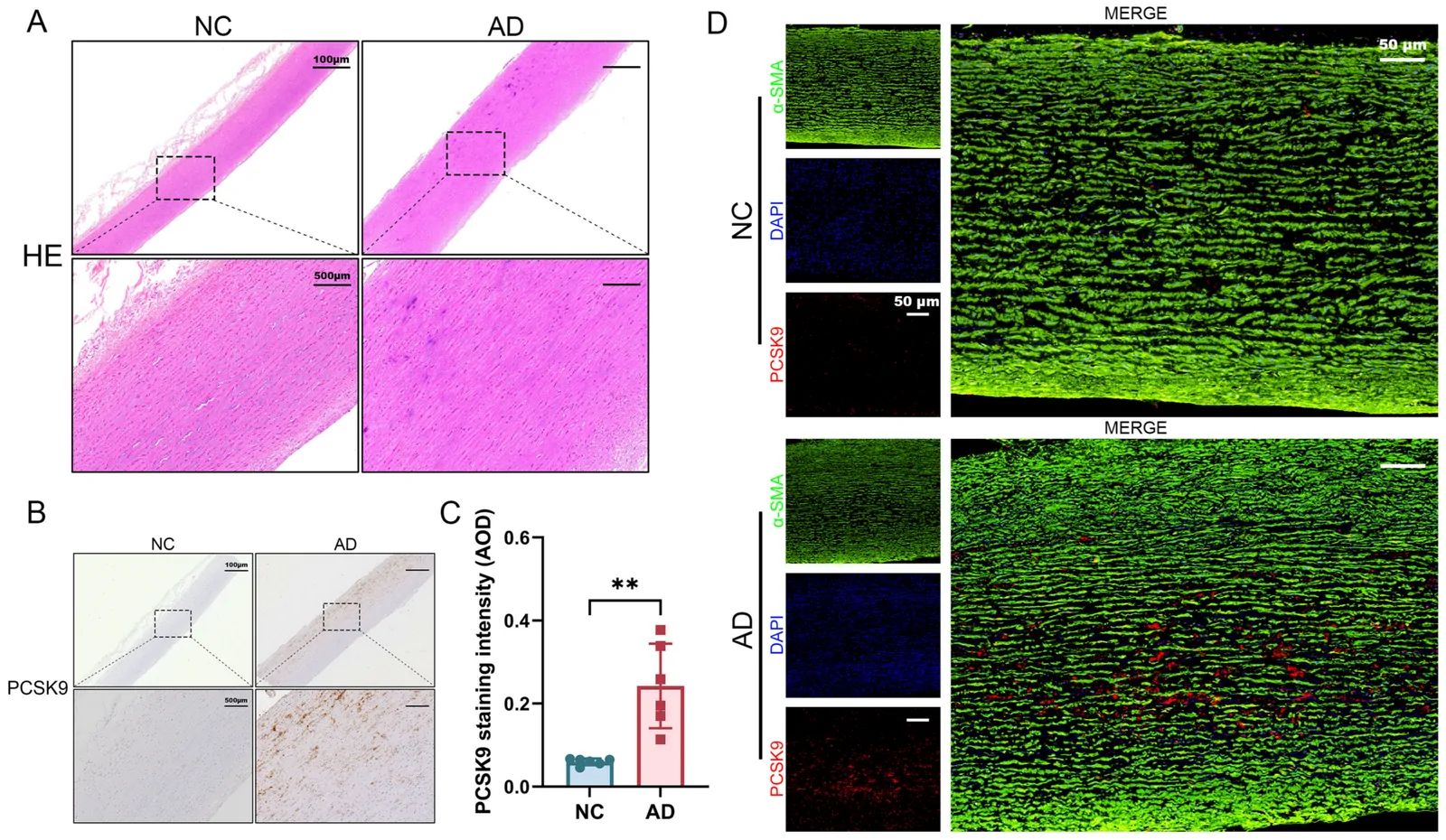
PCSK9 was enriched in aortic dissection tissues and localized to α-SMA-positive medial regions. (**A**) Representative hematoxylin and eosin (HE) staining images of NC and AD aortic tissue sections. (**B**) Representative immunohistochemical staining of PCSK9 in NC and AD aortic tissue sections. DAB-positive staining indicates PCSK9 immunoreactivity, and nuclei were counterstained with hematoxylin. Dashed boxes indicate the regions shown at higher magnification in the corresponding lower images. (**C**) Semi-quantitative analysis of PCSK9 immunohistochemical staining intensity (*n* = 6), expressed as average optical density (AOD). (**D**) Representative double immunofluorescence staining for PCSK9 and α-SMA in NC and AD aortic tissue sections. α-SMA is shown in green, PCSK9 in red, and nuclei in blue by DAPI staining. Merged images show the spatial relationship between PCSK9 and α-SMA-positive medial regions. Data are shown as mean ± SD, and each point represents an individual sample. Statistical comparisons were performed using an unpaired two-tailed Student’s *t*-test or Mann–Whitney U test, as appropriate. ** *p* < 0.01 vs. NC. Scale bars are indicated in the images. AD, aortic dissection; AOD, average optical density; DAB, 3,3′-diaminobenzidine; DAPI, 4′,6-diamidino-2-phenylindole; HE, hematoxylin and eosin; NC, normal control; α-SMA, alpha-smooth muscle actin.

To further characterize histological alterations and the spatial distribution of PCSK9 in human aortic tissues, hematoxylin and eosin staining, immunohistochemistry, and immunofluorescence co-staining were performed. Hematoxylin and eosin staining showed relatively preserved and organized aortic wall architecture in NC tissues, whereas AD tissues exhibited altered medial architecture and more disorganized tissue morphology, consistent with local structural remodeling of the dissected aortic wall (Figure 3A).

Immunohistochemical staining was performed to evaluate PCSK9 protein distribution in aortic tissue sections. PCSK9 immunoreactivity was weak in NC aortic tissues but was markedly increased in AD tissues, where stronger DAB-positive staining was observed within the aortic wall, particularly in the medial layer (Figure 3B). Semi-quantitative analysis further showed that PCSK9 staining intensity, expressed as the average optical density, was significantly higher in the AD group than in the NC group (0.242 ± 0.102 vs. 0.060 ± 0.008; *p* = 0.0014; Figure 3C), supporting increased local PCSK9 accumulation in human aortic dissection tissues.

The immunohistochemical staining results indicate that in the aortic tissue of patients with aortic dissection, PCSK9 is more abundantly expressed in the middle layer of the arterial wall. To further assess the spatial relationship between PCSK9 and vascular smooth muscle cell-rich regions, double immunofluorescence staining for PCSK9 and α-SMA was performed. In NC tissues, PCSK9 fluorescence was relatively limited, whereas α-SMA-positive medial structures were clearly observed. In AD tissues, PCSK9 fluorescence was increased and showed partial overlap with α-SMA-positive areas (Figure 3D).

## 4. Discussion

In this study, we used an integrated translational strategy combining public human aortic tissue transcriptomics, serum proteomic profiling in a TEVAR remodeling-stratified TBAD cohort, and multi-level validation in human serum and aortic tissues. This approach identified PCSK9 as a candidate molecule associated with both aortic dissection and unfavorable aortic remodeling after TEVAR. Among the seven overlapping molecules shared by the aortic transcriptomic and serum proteomic datasets, PCSK9 was the only candidate showing concordant upregulation in both diseased aortic tissue and serum samples from patients with negative remodeling. PCSK9 has established relevance to vascular biology beyond lipid metabolism, and recent genetic and experimental evidence has further implicated PCSK9 in aortic aneurysmal disease and vascular smooth muscle cell-related injury [11,13,29]. Subsequent validation confirmed that PCSK9 was elevated in serum, upregulated at the mRNA and protein levels in human aortic dissection tissues, and enriched in the dissected aortic wall, particularly in α-SMA-positive medial regions. Together, these findings suggest that PCSK9 may represent a candidate molecule linking circulating proteomic alterations with local pathological remodeling of the aortic wall.

The main strength of this study lies in the integration of complementary molecular layers. Aortic dissection and post-TEVAR remodeling are complex processes involving local wall degeneration, persistent false lumen perfusion, inflammation, extracellular matrix disruption, and systemic biological responses [30,31]. A single tissue-based or blood-based discovery strategy may therefore capture only part of the disease process. In this context, transcriptomic analysis of human aortic tissue provides information on local disease associated molecular programs, whereas serum proteomics reflects circulating proteins potentially associated with postoperative remodeling phenotypes and peripheral detectability. By intersecting 1534 differentially expressed genes from aortic tissue with 82 differentially abundant serum proteins from the remodeling-stratified TEVAR cohort, we reduced a broad discovery space to seven shared candidates. Although the seven-molecule overlap was not statistically enriched beyond chance, the intersection served as an exploratory strategy to reduce the candidate space and identify molecules showing cross-omics directional consistency. PCSK9 was prioritized because it was the only shared molecule showing concordant upregulation across both layers and because of its biological plausibility as a vascular remodeling-associated molecule. This cross-omics strategy therefore prioritized PCSK9 as a candidate potentially linking local aortic pathology with a measurable circulating signal. The other overlapping molecules may also reflect biologically relevant aspects of aortic dissection and postoperative remodeling. SERPINA1, FGA, ORM1, and S100A7 showed decreased transcript abundance in aortic tissue but increased abundance in serum. These discordant patterns may reflect differences between local aortic-wall alterations and systemic responses, including acute-phase responses, coagulation, inflammation, protein secretion, or altered circulating protein turnover. In contrast, APOA1 and IGHM were decreased in both datasets and may be related to altered lipid transport and humoral immune processes, respectively. Importantly, discordant patterns between tissue transcript abundance and circulating protein levels do not necessarily represent biological contradictions, because the transcriptomic and proteomic datasets represent different biological compartments and were derived from different patient populations. Moreover, circulating protein abundance may be influenced by systemic production, secretion, and protein turnover and therefore may not directly parallel local aortic-wall transcription. As these molecules were not independently validated in the present study, their biological interpretations remain hypothesis-generating and warrant further investigation.

PCSK9 is best known for promoting LDL receptor degradation and regulating cholesterol metabolism [32,33]. However, growing evidence indicates that PCSK9 also participates in vascular inflammation, endothelial dysfunction, oxidative stress, macrophage activation, and vascular remodeling [34,35]. These non-lipid effects are particularly relevant to aortic dissection, in which the central pathological features are not simple lipid deposition but medial degeneration, vascular smooth muscle cell dysfunction, inflammatory infiltration, and extracellular matrix breakdown. Large-scale genetic work in abdominal aortic aneurysm has identified pathways involving lipid metabolism, vascular remodeling, extracellular matrix dysregulation, and inflammation, and has highlighted PCSK9 inhibition as a potential therapeutic direction in aneurysmal disease [36]. More recent experimental studies have also suggested that PCSK9 in vascular smooth muscle cells may contribute to ferroptosis, senescence, apoptosis, inflammation, and aneurysm progression [37,38,39,40]. Although abdominal aortic aneurysm and aortic dissection are distinct disease entities, they share important pathological themes, including medial injury, inflammation, and matrix remodeling. Our finding that PCSK9 is increased in human aortic dissection tissues is therefore consistent with the broader concept that PCSK9 may participate in pathological remodeling of the aortic wall.

The serum proteomic findings further suggest potential clinical relevance in the setting of TEVAR. TEVAR promotes favorable remodeling by sealing the proximal entry tear and reducing false lumen pressurization, but a subset of patients still develop persistent false lumen flow, distal aortic expansion, and negative remodeling. Current risk assessment relies heavily on CTA based morphological features and longitudinal imaging follow-up. Recent studies and meta-analyses continue to emphasize the prognostic importance of remodeling patterns, false lumen status, distal aortic expansion, and inflammatory indices after TEVAR [41,42,43]. However, reliable circulating biomarkers that can complement imaging-based follow-up remain limited [15,44]. In this study, serum PCSK9 was elevated in the negative remodeling proteomic discovery group and was also increased in an independent AD serum validation cohort. These data suggest that circulating PCSK9 may have potential value as a remodeling-associated biomarker. This interpretation should remain cautious, because the current proteomic analysis was discovery-oriented and not powered to establish independent predictive performance. Future studies should evaluate whether PCSK9 improves risk stratification when combined with CTA morphology, false lumen characteristics, inflammatory markers, lipid parameters, and clinical variables.

The tissue validation results provide an additional layer of biological context. The aortic media is central to the initiation and progression of dissection, and vascular smooth muscle cells are essential for maintaining medial architecture and extracellular matrix homeostasis [7,9,45]. In the present study, immunohistochemistry showed stronger PCSK9 staining in AD tissues than in non-dissected control aortic tissues, with enrichment in the medial layer. Immunofluorescence further demonstrated partial spatial overlap between PCSK9 and α-SMA-positive regions, suggesting that increased PCSK9 expression is associated with VSMC-rich medial remodeling areas. This finding supports a local pathological relevance of PCSK9 within the dissected aortic wall. Nevertheless, co-localization does not prove that PCSK9 is produced by VSMCs, nor does it establish a direct regulatory effect on VSMC phenotype. The observed spatial association should therefore be interpreted as hypothesis-generating evidence that PCSK9 may be involved in, or reflect, medial remodeling in human aortic dissection.

Several potential mechanisms may explain the association between PCSK9 and aortic wall remodeling. PCSK9 has been linked to inflammatory signaling, including pathways related to TLR4/NF-κB activation and NLRP3 inflammasome regulation [12,13,14,46]. These pathways are relevant to vascular injury because they can amplify inflammatory responses, promote cytokine release, and contribute to matrix degradation. PCSK9 may also influence VSMC behavior through oxidative stress, ferroptosis, apoptosis, senescence, or phenotypic switching, mechanisms that have been reported in recent vascular and aneurysm-related experimental studies. In addition, serum PCSK9 may derive predominantly from hepatic secretion, whereas local aortic wall PCSK9 expression may represent a tissue-specific response to vascular injury [47,48]. However, these mechanisms were not directly investigated in the present study and should therefore be regarded as potential explanations based on previous studies rather than experimentally validated mechanisms in human aortic dissection. Our data support both circulating elevation and local aortic upregulation, but they cannot determine the relative contribution of hepatic versus vascular sources to serum PCSK9 levels. Functional studies using VSMCs, macrophages, endothelial cells, and animal models of dissection or aneurysmal remodeling will be required to clarify whether PCSK9 is a mediator of disease progression or mainly a marker of vascular injury.

The translational implications of these findings are also worth considering. PCSK9-targeting therapies, including monoclonal antibodies and small interfering RNA-based approaches, are already used clinically for lipid lowering and cardiovascular risk reduction [49,50,51]. If PCSK9 is confirmed to participate in aortic inflammation or medial remodeling, PCSK9 inhibition may have potential relevance beyond conventional lipid control. However, the present study does not provide evidence that PCSK9 inhibition can prevent aortic dissection, improve TEVAR remodeling, or modify aortic wall pathology. Such therapeutic implications remain speculative and require preclinical intervention studies, prospective observational cohorts, and eventually clinical trials specifically designed around aortic disease endpoints.

This study has several limitations. First, the proteomic discovery cohort was relatively small, and the findings require validation in larger, independent TBAD cohorts with standardized imaging follow-up. Second, the public aortic tissue transcriptomic dataset and the serum proteomic cohort were derived from different populations, which may introduce cohort heterogeneity despite improving external discovery breadth. Third, the human tissue validation cohort was clinically and anatomically distinct from the TEVAR-treated TBAD cohort. Therefore, these tissue findings support PCSK9 dysregulation in human aortic dissection but should not be interpreted as direct tissue-level evidence of PCSK9 alterations in the descending thoracic aorta of TEVAR-treated TBAD patients. Fourth, the current study is primarily associative and validation-oriented; it cannot establish causality between PCSK9 and aortic dissection or negative remodeling after TEVAR. Fifth, although serum, qPCR, Western blotting, immunohistochemistry, and immunofluorescence provided multi-level validation, functional experiments were not performed. Finally, the predictive value of circulating PCSK9 for postoperative remodeling was not assessed in a prospective model. Future work should test PCSK9 in larger longitudinal cohorts and determine whether it adds value to existing CTA-based and inflammatory risk models [52,53].

## 5. Conclusions

This study identifies PCSK9 as a cross-omics prioritized candidate molecule associated with aortic dissection and negative remodeling after TEVAR. The consistent elevation of PCSK9 in serum, aortic tissue mRNA and protein, together with its enrichment in α-SMA-positive medial regions, supports its potential role as a link between circulating proteomic alterations and local aortic wall pathology. These findings provide a rationale for further investigation of PCSK9 as a remodeling-associated biomarker and a possible mediator of vascular remodeling in TBAD.

## Figures and Tables

**Table 1:** Shared differentially expressed genes/proteins identified by cross-omics integration.

Molecule	Transcriptomics (AD vs. Control)	Serum Proteomics (P vs. S)	Direction (Transcriptomics/Proteomics)	Functional Implication
SERPINA1	log2FC = −2.167; *p* = 0.0053	FC = 1.332; *p* = 0.0176	↓/↑	Inflammation; protease inhibition
APOA1	log2FC = −1.731; *p* = 0.0071	FC = 0.603; *p* = 0.0057	↓/↓	Lipid metabolism
IGHM	log2FC = −2.210; *p* = 0.0146	FC = 0.571; *p* = 0.0402	↓/↓	Humoral immunity; immunoglobulin
FGA	log2FC = −1.913; *p* = 0.0035	FC = 1.505; *p* = 0.0211	↓/↑	Coagulation
ORM1	log2FC = −1.020; *p* = 0.0115	FC = 1.679; *p* = 0.0284	↓/↑	Acute-phase response
S100A7	log2FC = −1.742; *p* = 0.0141	FC = 2.092; *p* = 0.0065	↓/↑	Inflammation; immune response
PCSK9	log2FC = 1.050; *p* = 0.0038	FC = 1.244; *p* = 0.0406	↑/↑	Lipid metabolism; vascular remodeling

**Notes:** AD, aortic dissection; Control, non-dissected control aortic tissues; P, progression group with negative aortic remodeling after TEVAR; S, stable group with stable or positive aortic remodeling after TEVAR; FC, fold change. Direction is shown in the order of transcriptomics/proteomics. ↑ indicates upregulation, and ↓ indicates downregulation. The order of the molecules does not represent a statistical or biological ranking.

## Data Availability

The publicly available transcriptomic dataset analyzed in this study is available in the Gene Expression Omnibus (GEO) database under accession number GSE190635. The additional data generated and analyzed during the current study are available from the corresponding authors upon reasonable request, subject to applicable ethical and privacy restrictions.

## Supplementary Materials

The following supporting information can be downloaded at: https://www.mdpi.com/article/10.3390/jcdd13090442/s1, Figure S1: Uncropped Western blot images; Table S1: Differentially expressed genes identified in the GSE190635 dataset; Table S2: Clinical characteristics of the proteomic discovery cohort; Table S3: Differentially abundant serum proteins identified between the progression and stable groups.
