# Supplementary material for: Cross-Omics Identification and Validation of PCSK9 as a Candidate Molecule Associated with Aortic Dissection and Negative Remodeling After TEVAR
Source: J Cardiovasc Dev Dis. 2026 Sep 7;13(9):442. doi: 10.3390/jcdd13090442 (PMC13607724; doi:10.3390/jcdd13090442)
Supplement: Supplementary file 1 [file jcdd-13-00442-s001.zip › Supplementary Table S2. Clinical characteristics of the proteomic discovery cohort..pdf]

Supplementary Table S2. Clinical characteristics of the proteomic discovery cohort.

Variable	P group (n = 6)	S group (n = 6)	p value
Age, years	53.7 ± 12.3	56.0 ± 10.3	0.729
Male sex, n (%)	6 (100)	6 (100)	1.000
BMI, kg/m²	22.7 ± 2.1	25.8 ± 3.9	0.215
Systolic blood pressure, mmHg	140.2 ± 11.8	140.2 ± 13.0	1.000
Diastolic blood pressure, mmHg	73.3 ± 8.7	79.0 ± 16.4	0.476
Hypertension, n (%)	4 (67)	6 (100)	0.455
Diabetes mellitus, n (%)	0 (0)	0 (0)	1.000
Coronary artery disease, n (%)	1 (17)	0 (0)	1.000
Hyperlipidemia, n (%)	1 (17)	1 (17)	1.000
Statin use, n (%)	1 (17)	1 (17)	1.000
Preoperative hs-CRP, mg/L	16.8 ± 8.0	18.6 ± 7.9	0.698
Smoking history, n (%)	1 (17)	1 (17)	1.000
Drinking history, n (%)	0 (0)	0 (0)	1.000

Notes: Data are presented as mean ± SD or n (%). P group, progression group with negative aortic remodeling after TEVAR; S group, stable group with stable or positive aortic remodeling after TEVAR; BMI, body mass index; TEVAR, thoracic endovascular aortic repair; hs-CRP, high-sensitivity C-reactive protein Continuous variables were compared using an unpaired two-tailed Student's t-test, and categorical variables were compared using Fisher's exact test. Only available preoperative clinical variables are reported. Given the small sample size, the data are presented descriptively and should not be interpreted as evidence of balance or absence of confounding.
